# Synthesis, Characterization, Anti-Inflammatory and *in Vitro *Antimicrobial Activity of Some Novel Alkyl/Aryl Substituted Tertiary Alcohols

**DOI:** 10.3390/molecules161210337

**Published:** 2011-12-14

**Authors:** Muhammad Baseer, Farzana Latif Ansari, Zaman Ashraf, Rafiuzzaman SaeedulHaq

**Affiliations:** 1 Department of Chemistry, Quaid-i-azam University, Islamabad 45320, Pakistan; 2 Department of Chemistry, Allam Iqbal Open University, Islamabad 44000, Pakistan; 3 Riphah Institute of Pharmaceutical Sciences, Islamabad 44000, Pakistan

**Keywords:** tertiary alcohols, anti-inflammatory activity, antibacterial activity, antifungal activity

## Abstract

The synthesis of some novel alkyl/aryl substituted tertiary alcohols was accomplished in two steps. The synthetic route involves preparation of Grignard reagents by treating alkyl/aryl bromides with magnesium turnings in dry ether. Then substituted chalcones were reacted with the Grignard reagents to afford alkyl/aryl substituted tertiary alcohols **1-10**. The structures of the synthesized compounds were assigned on the basis of FT-IR, ^1^H-NMR, ^13^C-NMR and mass spectroscopic data. The *in vivo* anti-inflammatory activity of the synthesized compounds was evaluated using the carrageenan-induced hind paw edema method and was compared with that of ibuprofen. Some of the newly synthesized compounds showed promising anti-inflammatory activity. The tertiary alcohols **1-10** were also screened for antibacterial activity against ten bacterial strains using seven Gram-positive and three Gram-negative bacteria and for antifungal activity against *Aspergillus Flavus*, *Aspergillus Niger *and *Aspergillus pterus.* Tertiary alcohols **1-10** were found to exhibit good to excellent antimicrobial activities compared to levofloxacin and fluconazole used as standard drugs.

## 1. Introduction

The emergence of multiple-drug resistance organisms, such as methicillin-resistant *Staphylococcus aureus *and vancomycin resistant *Enterococci*, has created a major concern in the medical field and an urgent need for new antibacterial agents [1].

It is known that hydroxyl groups, amino groups and aromatic rings are general and particularly important functionalities in biologically active compounds [2]. With this in mind, we expected that tertiary alcohols with aromatic rings attached to a quaternary carbon atom should display biological activities, since these important functionalities should facilitate the interactions with the relevant receptor molecules [3]. Along these lines the potent broad-spectrum antifungal activities of two new enantiopure tertiary alcohols having fluoro-substituted aromatic rings has been reported [4].

A new class of compounds with a shielded tertiary alcohol in the transition state mimicking scaffold, showed high enzyme inhibition activities and excellent permeation through a Caco-2 cell membrane [5]. Some tertiary alcohol derivatives are good HIV-1 protease inhibitors [6], and active in the mouse writhing and rat tail-flick analgesic assay [7]. Tertiary alcohol derivatives exhibit thromboxane A_2_ and prostaglandin H_2_ receptor inhibition and are used for the treatment of a number of disorders such as coronary vasospasm, asthma and peptic ulcers [8,9]. Tertiary butyl alcohol can also be applied in the preparation of a hydrophobic drug-hydroxypropyl B-cyclodextrin complex of ketoprofen and nitrendipine, increasing the solubility of the drugs in both simulated gastric juice and in simulated intestinal fluid, which improves the absorption and pharmacodynamic properties of drugs [10].

Diarylquinolines, belonging to the quinoline class of compounds, possess a central heterocyclic quinolinic nucleus and side chains with tertiary alcohol and tertiary amine groups which are responsible for their antimycobacterial action [11]. β-Amino alcohols are a large class of compounds with a wide range of bioactivities, such as antiplasmodial [12], antileishmanial [13] and antiproliferative [14].

The proven efficiency of these compounds prompted us to synthesize some novel alkyl/aryl substituted tertiary alcohol derivatives. Tertiary alcohols can be synthesized by a number of synthetic routes, using (η^6^-fluoroarene)Cr(CO)_3_ complexes as a substrate [15], by oxidizing tertiary aldehydes with oxygen at elevated temperatures [16], while enantiomerically pure tertiary alcohols are synthesized in reactions catalyzed by enzymes [17] or by using the inexpensive asymmetric organic catalyst L-proline [18,19]. In this study the tertiary alcohols were synthesized using the Grignard reaction, which is a typical method for tertiary alcohol preparation [20]. The anti-inflammatory, antibacterial and antifungal activities of the newly synthesized compounds are also discussed in this paper.

## 2. Results and Discussion

**Scheme 1:**
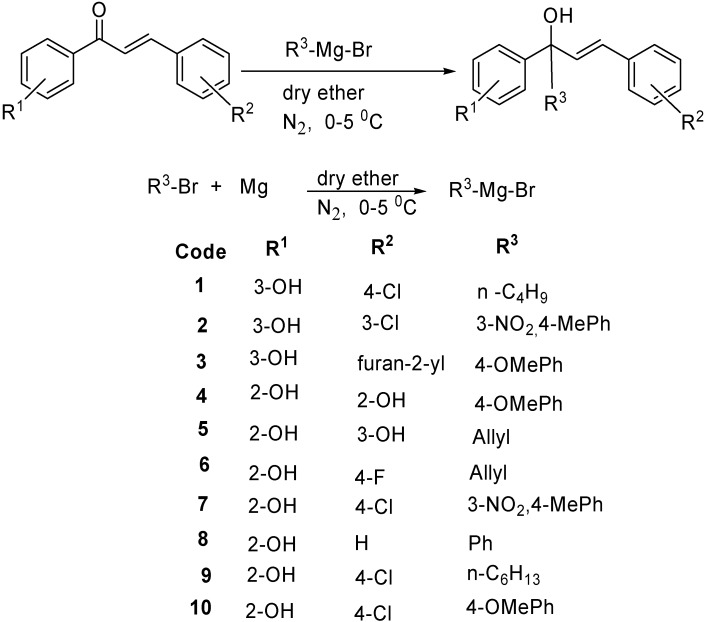
Synthesis of tertiary alcohols **1-10**.

We undertook these studies in order to prepare a novel series of tertiary alcohols to evaluate their anti-inflammatory and antimicrobial activities. The synthetic sequence leading to the alkyl/aryl substituted tertiary alcohols is outlined in Scheme 1. The alkyl/aryl bromides were treated with magnesium turnings in dry ether to afford the corresponding Grignard reagents. The chalcone derivatives were prepared in a single step by the Claisen-Schmidt condensation of 3-hydroxy-acetophenone with suitably substituted benzaldehydes; the synthesis and characterization data has been reported earlier [21]. The tertiary alcohols **1-10** were synthesized by the nucleophilic addition of freshly prepared Grignard reagents to the carbonyl carbon of the suitably substituted chalcones [22]. The physical constants of the synthesized tertiary alcohols **1-10** are presented in the Experimental section.

Typically, tertiary alcohols **1-10** are characterized by IR absorptions at 3302–3345 cm^−1^ for alcoholic hydroxyl and at 1632–1636 cm^−1^ for (CH=CH), respectively. The characteristic one proton singlets at *ca*δ 3.65 for (alcoholic O-H), two doublets at δ 7.43 for (=CH-Ar) and 7.81 ppm for (-CO-CH=), and peaks at *ca* 76 for respective carbons attached to alcoholic hydroxyl were also observed in the ^1^H- and ^13^C-NMR spectra, respectively. Mass spectra of the compounds showed [M^+^] peaks, which were in agreement with their molecular formulae.

### 2.1. Antiinflammatory Activity

The newly synthesized compounds were evaluated for their *in vivo* antiinflammatory activity using the carrageenan-induced hind paw edema method [23]. Adult Sprague-Dawley rats, weighing 150–200 g, were used. The animals were allowed food and water *ad libitum*, except during the experiment. They were housed in a room at 23 ± 2 °C with a 12 h light/dark cycle. The animals were randomly allocated into groups of six animals each at the beginning of the experiment and were fasted for 24 h before the experiment with free access to water. All of the compounds and the reference drug were suspended in 0.5% carboxymethyl cellulose (CMC) solution. The standard drug ibuprofen was administered orally at a dose of 20 mg/kg. The tested compounds were administered orally at an equimolar oral dose relative to 20 mg/kg of ibuprofen. The control group received a 0.5% CMC solution. Into the subplantar region of the right hind paw of each rat, 0.1 mL of 1% carrageenan solution in saline was injected subcutaneously, 1 h after the administration of the test compounds and standard drug. The right hind paw volume was measured after 3 h of carrageenan treatment by means of a plethysmometer. The percent edema inhibition was calculated from the mean effect in the control and treated animals according to the following equation:

Percent edema inhibition = (v_c_ − v_t_ / v_c_) × 100

where v_t_ represents the mean increase in paw volume in rats treated with tested compounds and v_c_ represents the mean increase in paw volume in the control group of rats. The potency was calculated as regards the percentage of the change of the standard and tested compounds, as depicted in the Table 1.

**Table 1:** Anti-inflammatory activity of tertiary alcohols **1-10**.

Compounds	Edema volume ± S.E after 3 h	% Inhibition of inflammation ^a^ after 3 h	Potency ^b^
**1**	1.080 ± 0.030 *	49.76	0.68
**2**	1.133 ± 0.049 *	47.30	0.64
**3**	1.000 ± 0.025 *	53.48	0.73
**4**	0.966 ± 0.021 *	55.06	0.75
**5**	1.883 ± 0.074	12.41	0.17
**6**	1.833 ± 0.060	14.74	0.20
**7**	0.800 ± 0.036 *	62.79	0.86
**8**	0.900 ± 0.051 *	58.13	0.79
**9**	1.000 ± 0.025 *	53.48	0.73
**10**	1.067 ± 0.033 *	49.76	0.68
**Ibuprofen**	0.583 ± 0.060 *	72.88	1
**Control**	2.150 ± 0.056	-	-

* Significance from control, P < 0.01; ^a^ Percent edema inhibition was calculated with regards to the control group; ^b^ Potency was calculated with regards to the percentage inhibition of the ibuprofen-treated group.

The synthesized compounds showed anti-inflammatory activity ranging from 12.41% to 62.79%, whereas the standard drug ibuprofen showed 72.88% inhibition of edema after three hours. The anti-inflammatory activity results revealed that the activity was dependant on the type and position of the functional group on the homocyclic and heterocyclic rings. Compound **7**, which possesses a polar hydroxyl functional group and an electronegative chloro substituent on the phenyl ring showed excellent results. Alcohol **9** possessing the same functionalities, but lacking the phenyl ring exhibited moderate edema inhibition compared to the standard drug. 

### 2.2. Antibacterial Activity

*In vitro* evaluation of antibacterial activity of the tertiary alcohols **1-10** was carried out against seven Gram negative bacterial strains *viz. Proteus mirabilis*, *Escherichia coli*, *Pseudomonas putida*, *Pseudomonas aeruginosa*, *Salmonalla typhi*, *Shigella flexineri *and *Klebsiella pneumoniae* and against three Gram positive bacterial strains: *Bacillus subtilis*, *Staphylococcus aureus *and *Micrococcus luteus*, by the agar well diffusion method [24]. Assays were conducted using Mueller Hinton Agar (MHA). Fresh inoculums of these strains were prepared and diluted with sterilized normal saline. The turbidity of these cultures was adjusted to 0.5 Mc-Farland. A uniform bacterial lawn was developed by sterile cotton swabs. 6 mm sized borer was used to make the wells in the inoculated plates. One mg of each sample was dissolved in DMSO (1.0 mL). Levofloxacin was used as positive control in this antimicrobial study. Levofloxacin (1.0 mg/mL), a broad spectrum antibiotic effective against a number of Gram positive and Gram negative bacterial strains, was used as standard. These plates were incubated at 37 °C for 24 h. Antibacterial activity of the tertiary alcohols **1-10** was determined by measuring the diameter of zone of inhibition (mm, ± standard deviation) and presented by subtracting the activity of the control. The tests were repeated three times and the results are reported as means of at least three determinations and the results are summarized in Table 2. The figures represent the zone of inhibition in millimeters.

**Table 2:** Antibacterial bioassay screening of tertiary alcohols **1-10**.

Codes	*P.m.*	*B.s.*	*E.c.*	*S a.*	*P. p.*	*P. a.*	*S. t.*	*M.l.*	*S. f.*	*K.p.*
**1**	14	04	10	10	08	10	07	04	05	09
**2**	10	-	12	09	10	13	11	08	11	12
**3**	17	08	11	-	13	11	13	10	09	14
**4**	16	05	10	15	15	-	10	-	11	10
**5**	14	-	14	07	11	14	14	09	14	15
**6**	10	-	11	08	10	15	10	10	10	11
**7**	24	06	13	10	24	22	16	16	23	15
**8**	21	03	12	09	21	24	19	-	21	17
**9**	16	10	14	12	16	15	16	14	16	17
**10**	14	08	12	-	14	12	14	08	14	16
**Standard**	30	20	30	25	30	28	30	25	30	30

Activity is presented in millimeters (mm), (**-**) No activity; *Pasteurella multocida (**P.m.**)*, *Bacillus subtilis (**B.s.**)*, *Escherichia coli (**E.c.**)*, *Staphylococcus aureus (**S.a.**)*, *Pseudomonas putida (**P.p.**)*, *Pseudomonas aeruginosa (**P.a.**)*, *Salmonella typhi (**S.t.**)*, *Micrococcus luteus (**M.l.**)*, *Shigella flexineri (**S.f.**)* and *Klebsiella pneumonae (**K.p.**)*.

The antibacterial activity results showed that tertiary alcohols **1-10** have higher antibacterial potential against the tested Gram negative bacterial strains than the Gram positive ones. Compounds **7** and **8** exhibited excellent results against *Proteus mirabilis*, *Pseudomonas putida*, *Pseudomonas aeruginosa* and *Shigella flexineri*. The position and nature of the substituent is critical for activity. Compound **7** possesses polar hydroxyl, chloro and nitro groups on three phenyl rings while alcohol **8** possess one hydroxyl-substituted and two unsubstituted phenyl rings. In compound **7** the polarity of the hydroxyl group and electronegativity of the halogen play an important role in antibacterial activity while the planarity of the two unsubstituted phenyl rings in alcohol **8** is also significant. All of the remaining alcohols showed moderate activity against selected bacteria.

### 2.3. Antifungal Activity

*In vitro *antifungal activity of the tertiary alcohols **1-10** was tested against three fungi; *Aspergillus flavus*, *Aspergillus nigar and Aspergillus pterus *using the poison plate method [25]. Potato dextrose agar (PDA) plates were prepared by using the pour plate technique for each compound. A 2% concentration of the synthesized compounds in DMSO as a solvent was used. A 2% solution of fluconazole was used as standard. A drug free control was included and plates were observed for growth after 48 h of static incubation at 30 °C and results are presented in Table 3.

**Table 3:** Antifungal bioassay screening of tertiary alcohols **1-10**.

Codes		*Aspergillus flavus*	*Aspergillus niger*		*Aspergillus pterus*
**1**		38	26		41
**2**		11	05		13
**3**		09	-		10
**4**		15	06		15
**5**		19	09		11
**6**		31	20		33
**7**		18	-		15
**8**		16	11		17
**9**		17	-		14
**10**		29	18		30
**Standard**		37	23		36

Activity is presented in millimeters, (-) No activity.

Among all the synthetic alcohols **1-10** compounds **1**, **6** and **10** are more active than the remaining ones. The excellent results were shown by compound **1**, which exhibited higher growth inhibition than the standard drug. It possesses 3-hydroxy- and 4-chloro-substituted phenyl rings along with the presence of an *n*-butyl alkyl chain. Compounds **6** and **10** also showed good antifungal activity; both of them possess polar hydroxyl- and electronegative halogen-substituted phenyl rings. The electronegativity of the halogens is important as the alcohol **6** with a more electronegative fluorine showed higher antifungal activity against *Aspergillus flavus*, *Aspergillus nigar and Aspergillus pterus* than compound **8**, which possesses a less electronegative chlorine. The electronegativity of the substituent is directly related to their antifungal activity. The presence of an allyl group is another structural feature which determines antifungal potential. The remaining analogues showed moderate to low antifungal activity.

## 3. Experimental

Melting points were recorded using a digital Gallenkamp (SANYO) model MPD.BM 3.5 apparatus and are uncorrected. ^1^H- and ^13^C-NMR spectra were determined in acetone-d_6_ at 300 MHz and 75 MHz, respectively, using a Bruker AM-300 model spectrophotometer. IR spectra were recorded on a Perkin Elmer Spectrum BX spectrophotometer as KBr pellets. Mass spectra (EI, 70eV) on a GC-MS instrument. Bioactivities were determined out at the Riphah Institute of Pharmaceutical Sciences, Riphah International University (Islamabad, Pakistan). All chemicals were purchased from Merck and Aldrich, and were used without further purification.

### 3.1. Synthesis of Tertiary Alcohols ***1-10***

#### 3.1.1. General Procedure

An equimolar mixture of magnesium turnings and the appropriate alkyl, alkenyl or substituted aryl bromide was vigorously stirred in the presence of dry ether at 0–5 °C under an inert atmosphere to afford the corresponding Grignard reagent. A solution of freshly prepared Grignard reagent (10 mmol) in dry ether (10 mL) was treated with substituted chalcone (10 mmol) at 0–5 °C under a continuous flow of N_2_ for 30 min. The reaction mixture was stirred overnight and the precipitates formed were filtered and recrystallized in ethanol to afford the tertiary alcohols **1-10**, whose structures were confirmed by their spectroscopic data.

*(E)-3-(1-(4-Chlorophenyl)-3-hydroxynon-1-en-3-yl)phenol* (**1**). Prepared from 3′-hydroxy-4-chloro-chalcone (1.29 g, 5.0 mmol) and *n*-hexyl magnesium bromide (0.94 g, 5.0 mmol). Yield 78%; m.p. 167–168 °C; FTIR υ_max_: 3,309 (O-H), 1,621 (C=C), 751 (C-Cl) cm^−1^; ^1^H-NMR δ 3.67 (s, 1H, OH-alcoholic), 6.67 (d, *J =* 15.8 Hz, 1H, H-C^α^ vinyl), 6.38 (d, *J =* 15.5 Hz, 1H, H-C^β^ vinyl), 9.1 (s, 1H, OH-phenolic), 6.87–7.69 (m, 8H, aromatic-H); ^13^C-NMR δ 14.1 (CH_3_), 22–45 (CH_2_), 81.3 (C-OH), 129.2 (β-C), 130.4 (α-C), 113–135 (aromatic-C); MS: *m/z* (%) 346.10 (M^+^+2, 32%), 344.10 (M^+^, 100%). 

*(E)-3-(3-(3-Chlorophenyl)-1-hydroxy-1-(4-methyl-3-nitrophenyl)allyl)phenol* (**2**). Prepared from 3′-hydroxy-3-chlorochalcone (1.29 g, 5.0 mmol) and 4-methyl-3-nitrophenylmagnesium bromide (1.195 g, 5.0 mmol). Yield 79%; m.p. 189–190 °C FTIR υ_max_: 3,302 (O-H), 1,619 (C=C), 753 (C-Cl) cm^−1^; ^1^H-NMR δ 3.62 (s, 1H, OH-alcoholic), 6.63 (d, *J =* 15.6 Hz, 1H, H-C^α^ vinyl), 6.37 (d, *J =* 15.4 Hz, 1H, H-C^β^ vinyl), 9.2 (s, 1H, OH-phenolic), 6.87–8.13 (m, 11H, aromatic-H); ^13^C-NMR δ 21.3 (CH_3_), 81.5 (OH), 129.2 (β-C), 130.3 (α-C), 114–139 (aromatic-C); MS: *m/z* (%) 397.10 (M^+^+2, 32%), 395.10 (M^+^, 100%).

*(E)-3-(3-(Furan-2-yl)-1-hydroxy-1-(4-methoxphenyl)allyl)phenol* (**3**). Prepared from 3′-hydroxy-3-(furan-2-yl)chalcone (1.07 g, 5.0 mmol) and 4-methoxyphenylmagnesium bromide (1.05 g, 5.0 mmol). Yield 67%; m.p. 249 °C; FTIR υ_max_: 3,316 (O-H), 1,632 (C=C) cm^−1^; ^1^H-NMR δ 3.67 (s, 1H, OH-alcoholic), 6.68 (d, *J =* 15.5 Hz, 1H, H-C^α^ vinyl), 6.39 (d, *J =* 15.3 Hz, 1H, H-C^β^ vinyl), 9.0 (bs, 1H, OH-phenolic), 6.52–7.30 (m, 11H, aromatic-H); ^13^C-NMR δ 80.2 (C-OH), 129.6 (β-C), 130.1 (α-C), 116–139 (aromatic C); MS: *m/z* (%) 324.10 (M^+^+2, 32%), 322.10 (M^+^, 100%). 

*(E)-2,2′-(3-Hydroxy-3-(4-methoxyphenyl)prop-1-ene-1,3-diyl)diphenol* (**4**). Prepared from 2,2′-dihydroxychalcone (1.2 g, 5.0 mmol) and 4-methoxyphenylmagnesium bromide (1.05 g, 5.0 mmol). Yield 74%; m.p. 231 °C; FTIR υ_max_: 3,345 (O-H), 1,629 (C=C) cm^−1^; ^1^H-NMR δ 3.66 (s, 1H, OH-alcoholic), 6.50 (d, *J =* 15.9 Hz, 1H, H-C^α^ vinyl), 6.65 (d, *J =* 15.8 Hz, 1H, H-C^β^ vinyl), 9.1 (bs, 1H, OH-phenolic), 3.85 (s, 3H, OCH_3_), 6.70–7.67 (m, 12H, aromatic-H); ^13^C-NMR δ 83.4 (C-OH), 123.2 (β-C), 126.1 (α-C), 113–138 (aromatic-C); MS: *m/z* (%) 384.10 (M^+^, 100%).

*(E)-2-(3-Hydroxy-1-(3-hydroxyphenyl)hexa-1,5-dien-3-yl)phenol* (**5**). Prepared from 3,2′-dihydroxy-chalcone (1.2 g, 5.0 mmol) and allyl magnesium bromide (0.72 g, 5.0 mmol). Yield 68%; m.p. 198–199 °C; FTIR υ_max_: 3,341 (O-H), 1,635 (C=C) cm^−1^; ^1^H-NMR δ 3.66 (s, 1H, OH-alcoholic), 6.67 (d, *J =* 15.0 Hz, 1H, H-C^α^ vinyl), 6.38 (d, *J =* 15.5 Hz, 1H, H-C^β^ vinyl), 9.1 (bs, 1H, OH-phenolic), 2.31–2.57 (dd, *J =* 8.1 Hz, 4.2 Hz, 2H, CH_2_), 5.01–5.08 (dd, *J =* 4.5 Hz, 4.6 Hz, 2H, CH_2_), 6.65–7.60 (m, 8H, aromatic-H); ^13^C-NMR δ 51.3 (CH_2_), 79.6 (C-OH), 129.2 (β-C), 130.4 (α-C), 116–139 (aromatic-C); MS: *m/z* (%) 282.10 (M^+^, 100%).

*(E)-2-(1-(4-Fluorophenyl)-3-hydroxyhexa-1,5-dien-3-yl)phenol* (**6**). Prepared from 2′-hydroxy-4-fluorochalcone (1.21 g, 5.0 mmol) and allyl magnesium bromide (0.84 g, 5.0 mmol). Yield 61%; m.p. 161.9 °C; FTIR υ_max_: 3,313 (O-H), 1,617 (C=C) cm^−1^; ^1^H-NMR δ 3.67 (s, 1H, OH-alcoholic), 6.67 (d, *J =* 15.0 Hz, 1H, H-C^α^ vinyl), 6.38 (d, *J =* 15.5 Hz, 1H, H-C^β^ vinyl), 9.1 (bs,1H, OH-phenolic), 2.31–2.57 (dd, *J =* 8.1 Hz, 4.2 Hz, 2H, CH_2_), 5.01–5.08 (dd, *J =* 4.5 Hz, 4.6 Hz, 2H, CH_2_), 6.66–7.67 (m, 8H, aromatic-H); ^13^C-NMR δ 51.5 (CH_2_), 77.2 (C-OH), 129.1 (β-C), 130.3 (α-C), 115–135 (aromatic-C); MS: *m/z* (%) 284.10 (M^+^, 100%).

*(2E)-[3-(4-Chlorophenyl)-1-hydroxy-1-(4-methyl-3-nitrophenyl)-allyl]-phenol* (**7**). Prepared from 4-chloro-2′-hydroxychalcone (1.29 g, 5.0 mmol) and 4-methyl-3-nitrophenyl magnesium bromide (1.195 g, 5.0 mmol). Yield 61%; m.p. 192–193 °C; FTIR υ_max_: 3,332 (O-H), 1,619 (C=C) cm^−1^; ^1^H-NMR δ 3.65 (s, 1H, OH-alcoholic), 6.68 (d, *J =* 15.3 Hz, 1H, H-C^α^ vinyl), 6.48 (d, *J =* 15.8 Hz, 1H, H-C^β^ vinyl), 9.1 (bs, 1H, OH-phenolic), 2.31 (s, 3H, CH_3_), 6.76–7.87 (m, 11H, aromatic-H); ^13^C-NMR δ 21.3 (CH_3_), 76.1 (C-OH), 129.3 (β-C), 130.2 (α-C), 114–139 (aromatic-C); MS: *m/z* (%) 395.10 (M^+^, 100%).

*(E)-2-(1-Hydroxy-1-3-diphenylallyl)phenol* (**8**). Prepared from 2′-hydroxychalcone (1.22 g, 5.0 mmol) and phenylmagnesium bromide (0.90 g, 5.0 mmol). Yield 59%; m.p. 211–212 °C; FTIR υ_max_: 3,322 (O-H), 1,609 (C=C) cm^−1^; ^1^H-NMR δ 3.65 (s, 1H, OH-alcoholic), 6.67 (d, *J =* 15.2 Hz, 1H, H-C^α^ vinyl), 6.47 (d, *J =* 15.7 Hz, 1H, H-C^β^ vinyl), 9.0 (s, 1H, OH-phenolic), 6.96–7.89 (m, 14H, aromatic-H); ^13^C-NMR δ 79.3 (C-OH), 129.1 (β-C), 130.4 (α-C), 118–133 (aromatic-C); MS: *m/z* (%) 302.10 (M^+^, 100%).

*(E)-2-(1-(4-Chlorophenyl)-3(hydroxyhexa-1-en-3-yl)phenol* (**9**). Prepared from 4-chloro-2′-hydroxy-chalcone (1.29 g, 5.0 mmol) and phenylmagnesium bromide (0.94 g, 5.0 mmol). Yield 63%; m.p. 197–198 °C; FTIR υ_max_: 3,325 (O-H), 1,605 (C=C) cm^−1^; ^1^H-NMR δ 3.66 (s, 1H, OH-alcoholic), 6.67 (d, *J =* 15.1 Hz, 1H, H-C^α^ vinyl), 6.47 (d, *J =* 15.4 Hz, 1H, H-C^β^ vinyl), 9.1 (s, 1H, OH-phenolic), 1.77–1.33 (dd, *J =* 8.4 Hz, 4.3 Hz, CH_2_), 0.89 (s, 3H, CH_3_), 6.76–7.85 (m, 8H, aromatic-H); ^13^C-NMR δ 21.4 (CH_3_) 78.1 (C-OH), 129.3 (β-C), 130.4 (α-C), 115–130 (aromatic-C); MS: *m/z* (%) 346.12 (M^+^+2, 32%), 344.10 (M^+^, 100%).

*(E)-2-(3-(4-Chlorophenyl)-1-(hydroxy-1-(4-methoxyphenyl)allyl)phenol* (**10**). Prepared from 4-chloro-2′-hydroxychalcone (1.29 g, 5.0 mmol) and anisyl magnesium bromide (1.05 g, 5.0 mmol). Yield 71%; m.p. 211–212 °C; FTIR υ_max_: 3,320 (O-H), 1,617 (C=C) cm^−1^; ^1^H-NMR δ 3.66 (s, 1H, OH-alcoholic), 6.67 (d, *J =* 15.1 Hz, 1H, H-C^α^ vinyl), 6.39 (d, *J =* 15.4 Hz, 1H, H-C^β^ vinyl), 9.1 (s, 1H, OH-phenolic), 3.85 (s, 3H, OCH_3_), 6.76–7.88 (m, 12H, aromatic-H); ^13^C-NMR δ 58.5 (OCH_3_) 70.2 (C-OH), 129.6 (β-C), 125.3 (α-C), 114–139 (aromatic-C); MS *m/z* (%) 368.12 (M^+^+2, 32%), 366.10 (M^+^, 100%).

## 4. Conclusions

We have investigated the effect of different substituents on growth inhibition of different bacterial and fungal strains and edema inhibition by some novel aryl/alkyl tertiary alcohols. The antifungal potential of the tertiary alcohols was high than the antibacterial activity. One of the tertiary alcohols showed higher antifungal activity than the standard. This derivative possesses a polar hydroxyl group, a hydrophobic alkyl chain and an electronegative chloro-substituent, which all play important role in antifungal activity.

## References

[1] Hong-Xi X., Song F.L. (2001). Activity of plant flavonoids against antibiotic-resistant bacteria. Phytother. Res..

[2] Patrick G.L. (2001). An Introduction to Medicinal Chemistry.

[3] Yasohara Y., Miyamoto K., Kizaki N., Hasegawa J. (2001). A practical chemoenzymatic synthesis of a key intermediate of antifungal agents. Tetrahedron Lett..

[4] Abdel-Rahman H.M., Al-Karamany G.S., El-Koussi N.A., Youssef A.F., Kiso Y. (2002). HIV protease inhibitors: Peptidomimetic drugs and future perspectives. Curr. Med. Chem..

[5] Rodriguez-Barrios F. (2004). HIV protease inhibition: Limited recent progress and advances in understanding current pitfalls. Curr. Top. Med. Chem..

[6] Xiongyu W., Per O., Jenny K.E., Johan U., Torsten U., Hans W., Bertil S., Anders H., Mars L. (2008). Tow-carbon-clongated HIV-1 protease inhibitors with a tertiary alcohol containing transition-state mimic. J. Med. Chem..

[7] Kotick M.P., Leland D.L., Polazzi J.Q., Schut R.N. (1980). Analgesic narcotic antagonists. 1.8. beta. alkyl-, 8. beta. -acyl-, and 8. beta. (tertiary alcohol)dihydrocodeinones and -dihydromorphinones. J. Med. Chem..

[8] Fiddler G.I., Lumley P. (1990). Preliminary clinical studies with thromboxane synthetase inhibitors and thromboxane receptor blockers. Circulation.

[9] Cross P.E., Dickinson R.P. (1987). Thromboxane synthetase inhibitors and antagonists. Annu. Rep. Med. Chem..

[10] Wang Z., Deng Y., Zang X. (2006). The novel application of tertiary butyl alcohol in the preparation of hydrophobic drug-HP & CD complex. J. Pharm. Pharmacol..

[11] Alberto M., Anna C.C., Kelly D.E., Afranio K. (2010). The first compound of a new class of potent anti-tuberculosis drugs. Chem. Future Microbiol..

[12] Hans R.H., Gut J., Rosenthal P.J., Chibale K. (2010). Comparison of the antiplasmodial and falcipain-2 inhibitory activity of β-amino alcohol thiolactone-chalcone and isatin-chalcone hybrids. Bioorg. Med. Chem. Lett..

[13] Coimbra E.S., Almeida D., Taveira A.F., de Costa C.F., de Almeida A.C., Reis E.F.C., da Silva A.D. (2010). Synthesis and antileishmanial activity of lipidic amino alcohols. Chem. Biol. Drug Des..

[14] Cordova I., Leon L.G., Leon F., Andres S.L., Luis J.G., Padron J.M. (2006). Synthesis and antiproliferative activity of novel sugiol β-amino alcohol analogues. Eur. J. Med. Chem..

[15] Costa R.G., Curto M.J., Furtado O.R. (2000). Novel synthesis of haloaromatic tertiary alcohols using (η^6^-fluoroarene)tricarbonylchromium(0) complexes. Synth. Commun..

[16] Merger F., Nestler G. (1985). Preparation of Tertiary Alcohols. U.S. Patent.

[17] Özdemirhan D., Sezer S., Sönmez Y. (2008). Enzyme-catalyzed resolution of aromatic ring fused cyclic tertiary alcohols. Tetrahedron: Asymmetry.

[18] Sakhthivel K., Notz W., Bui T., Barbas C.F. (2001). Amino acid catalyzed direct asymmetric aldol reactions: A bioorganic approach to catalytic asymmetric carbon-carbon bond forming reactions. J. Am. Chem. Soc..

[19] Bogevig A., Kumaragurubaran N., Jorgensen K.A. (2002). Direct catalytic asymmetric aldol reactions of aldehydes. Chem. Commun..

[20] Phrmaceuticals S.S. (2005). Synthesis of pharmaceutical intermediates aiming at construction of optically active tertiary alcohols as a key technology.

[21] Ansari F.L., Nazir S., Noureen H., Mirza B. (2005). Combinatorial synthesis and antibacterial evaluation of an indexed chalcone library. Chem. Biodivers..

[22] Haugan J.A. (1997). Total synthesis of C31-methyl ketone apocarotenoids 2: The first total synthesis of (3R)-triophaxanthin. Acta. Chem. Scand..

[23] Winter C.A., Risley E.A., Nuss G.W. (1962). Carrageenin-induced edema in hind paw of the rat as an assay for antiinflammatory drugs. Proc. Soc. Exp. Biol. Med..

[24] Okeke M.I., Iroegbu C.U., Eze E.N., Okoli A.S., Esimone C.O. (2001). Evaluation of extracts of the root of landolphia owerrience for antibacterial activity. J. Ethnopharmacol..

[25] Shastri R.V., Varudkar J.S. (2009). Synthesis and antimicrobial of 3-propen 1,2-benzisoxazole derivatives. Indian J. Chem. Sec..

